# Magneto-optical properties in IV-VI lead-salt semimagnetic nanocrystals

**DOI:** 10.1186/1556-276X-7-374

**Published:** 2012-07-07

**Authors:** Silvio J Prado, Leonardo Villegas-Lelovsky, Augusto M Alcalde, Victor Lopez-Richard, Gilmar E Marques

**Affiliations:** 1 Faculdade de Ciencias Integradas do Pontal, Universidade Federal de Uberlandia, Ituiutaba Minas Gerais 38302-000, Brasil; 2 Instituto de Fisica, Universidade Federal de Uberlandia; 3 Departamento de Fisica, Universidade Federal de Sao Carlos

**Keywords:** Nanocrystals, Quantum dots, DMS, II-VI semiconductors, Lead salts, Magneto-optical properties

## Abstract

We present a systematic study of lead-salt nanocrystals (NCs) doped with Mn. We have developed a theoretical simulation of electronic and magneto-optical properties by using a multi-band calculation including intrinsic anisotropies and magnetic field effects in the diluted magnetic semiconductor regime. Theoretical findings regarding both broken symmetry and critical phenomena were studied by contrasting two different host materials (PbSe and PbTe) and changing the confinement geometry, dot size, and magnetic doping concentration. We also pointed out the relevance of optical absorption spectra modulated by the magnetic field that characterizes these NCs.

## Review

Recently, the successful fabrication of IV-VI nanocrystals doped with Mn has shown possible effective tuning of the emission energy from infrared (dot radius ≃ 200 Å) up to near-ultraviolet (dot radius ≃ 20 Å) regions [1]. The IV-VI semiconductors, such as PbSe nanocrystals (NCs), provide access to the limit of strong quantum confinement where, besides the changes induced by very small dot size, the direct narrow band-gap that can also be engineered by the gradual addition of dilute amounts of magnetic Mn ions to the dot structure. The members of the lead-salt family, such as PbSe and PbTe, have rock-salt crystalline structure with a direct bandgap in the *L*-point and the energy branches are four-fold degenerate. The bottom of the conduction band has $L_{6}^{-}$ symmetry with the top of the valence band displaying $L_{6}^{+}$ symmetry of the double group *D*_3_. This corresponds to the opposite situation observed in III-V or II-VI zinc blend materials, since here the valence band-edge Bloch function displays s-like symmetry whereas the conduction band-edge Bloch function has p_*z*_-like symmetries, where *z* denotes the 〈111〉 direction of the cubic lattice [2].

In this letter, we contrast quantum dot electronic properties of two IV-VI semiconductor materials by modifying the quantum confinement from spherical to semispherical and varying the diluted concentration of incorporated Mn^2 + ^ ions. The electronic, magnetic, and optical properties are studied as a function of Mn content for varying temperature. The total Hamiltonian of the system is *H *=* H*_*kp*_ + *V* + *H*_*x*_ where *H*_*kp*_ is the hyperbolic or Kane-Dimmock [3]**k **·** p **Hamiltonian model for IV-VI semiconductors, *V * is a hard wall confinement potential and *H*_*x*_ is the exchange interaction between ^Mn2 + ^ ions and conduction band (valence band) spins. Here, *H*_*kp *_was slightly modified to explore spherical symmetries of the confinements

(1) $$H_{\text{kp}}=\left[\begin{array}{cccc} E_{g}-D_{1}^{-} & \frac{\hbar P_{l}}{m_{0}}P_{z} & 0 & \frac{\hbar P_{t}}{m_{0}}P_{-}\\ \frac{\hbar P_{l}}{m_{0}}P_{z} & D_{2}^{+} & \frac{\hbar P_{t}}{m_{0}}P_{-} & 0\\ 0 & \frac{\hbar P_{t}}{m_{0}}P_{+} & E_{g}-D_{1}^{-} & \frac{\hbar P_{l}}{m_{0}}P_{z}\\ \frac{\hbar P_{t}}{m_{0}}P_{+} & 0 & \frac{\hbar P_{l}}{m_{0}}P_{z} & D_{2}^{+} \end{array}\right]$$

where $D_{i}^{\pm }=-\frac{\hbar^{2}\nabla^{2}}{2m_{t}^{\pm }}+C_{i}P_{z}^{2}$, with ∇^2^ as the 3D Laplacian operator, and $C_{1}=\frac{\hbar^{2}}{2}\left(\frac{1}{m_{l}^{-}}-\frac{1}{m_{t}^{-}}\right)$$C_{2}=\frac{\hbar^{2}}{2}\left(\frac{1}{m_{l}^{+}}-\frac{1}{m_{t}^{+}}\right)$ are electron and hole effective mass terms while *P*_*t*_and *P*_*l*_ are the anisotropic conduction-valence Kane-Dimmock coupling parameters for longitudinal and transverse directions; *P*_*z*_ and *P*_±_ =* P*_*x *_±* iP*_*y*_ are the momentum operators, whereas *E*_*g *_is the bandgap and *m*_0_ is the free electron mass. The relevant Kane-Dimmock parameters for the materials analyzed in this work can be found in [4,5].

Also, *H*_*x *_= −* x*/2〈*S*_*z*_(*BT*)〉*N*_0_ ·* α*(·*β*), where 〈*S*_*z*_(*BTx*)〉 is the mean field magnetization at temperature *T*, represented as a Brillouin function in dilute doped sample containing *N*_0_ unit cells and Mn content, *x*[6]. Finally, *α* and *β* are the exchange constants for the semimagnetic materials, *N*_0_ ·* α *= −0.08 eV and *N*_0_·*β *= 0.02 eV for PbMnSe, while *N*_0_·*α *= −0.45 eV and *N*_0_·*β *= 0.29 eV for PbMnTe [5].

A complete set of eigenfunctions for the total Hamiltonian *H* can be spanned in terms of products of periodic Bloch functions |*J*,*J*_*z*_〉 near the *L*-point and envelope functions. For spherical confinement, we expand the four-component spinor wave functions in two Hilbert subspaces with the general form [7,8].

(2) $$\;|\psi_{I[\text{II}]}^{M}(r)\rangle =\underset{n}{\sum }\overset{\infty }{\underset{L\geq \left|M\right|}{\sum }}\left(\begin{array}{c} C_{n,2L[2L+1]}^{M}f_{n,2L[2L+1]}^{M}|L_{6}^{-}↑\rangle \\ C_{n,2L+1[2L]}^{M}f_{n,2L+1[2L]}^{M}|L_{6}^{+}↑\rangle \\ C_{n,2L[2L+1]}^{M+1}f_{n,2L[2L+1]}^{M+1}|L_{6}^{-}↓\rangle \\ C_{n,2L+1[2L]}^{M+1}f_{n,2L+1[2L]}^{M+1}|L_{6}^{+}↓\rangle \end{array}\right),$$

For the spherical model, these states fulfill the boundary condition $\Psi_{I,\text{II}}^{M}(R)=0$ at the dot radius; thus, the function components have the form $f_{n,L}^{M}(r,\theta ,\phi )=A_{n,L}j_{L}(k_{n}^{L}r)Y_{L}^{M}(\theta ,\phi )$ where *A*_*n*,*L*_ is a normalization constant, *j*_*L*_(*x*) is the spherical Bessel function, and $Y_{L}^{M}(\theta ,\phi )$ are the spherical harmonics. The subspaces must be constructed with special combinations of even ( $f_{n,L}^{M}(r)$) or odd ( $f_{n,2L+1}^{M}(r)$) with wave number $k_{n}^{L}=\mu_{n}^{l}/R$, where $\mu_{n}^{l}$ is the *n*th zero of *j*_*L*_(*x*) = 0. For the semispherical structures, the states must also fulfill the boundary condition $\Psi_{I,\text{II}}^{M}(r,\theta =\frac{\Pi }{2},\phi )=0$ at the equator plane which restricts the set of quantum numbers *L* and *M* to the condition |*L*−*M*| = odd number. Hence, the parities of the spinor components differ from the full spherical case and the states $|\psi_{I[\text{II}]}^{M}(r)\rangle$ for a semispherical confinement require the replacement 2*L* (2*L* + 1) in the second (third) line of Equation 2 by 2*L* + 1 (2*L*).

**Figure 1:**
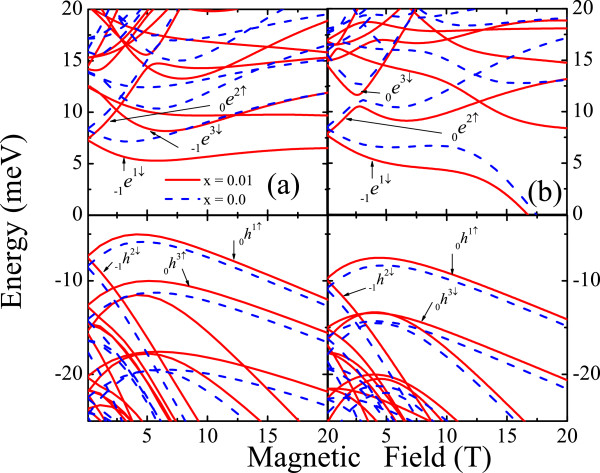
**Conduction and valence band energy levels as function of magnetic field in Pb**_**1−*x***_**Mn**_**x**_**Se NCs with spherical (a) and semispherical (b) confinements of radius R = 300 A and*T *= 1.8 K.** The subbands structure with (solid line) and without Mn-doping (dashed line) were calculated using *E*^*c*(*v*)^ −* Eg*(*x*).

**Figure 2:**
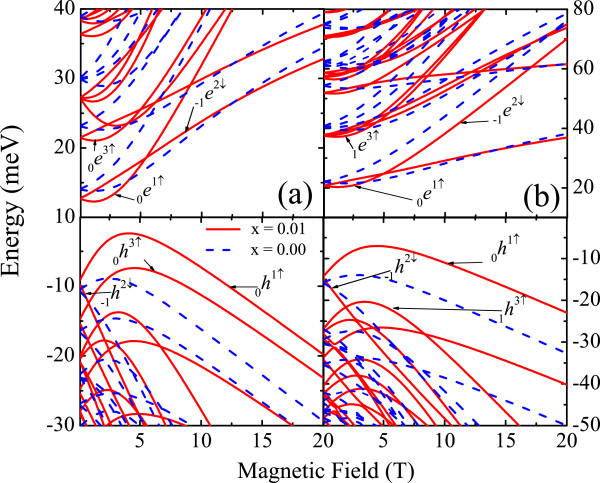
**Conduction and valence band energy levels as function of the magnetic field in Pb**_**1−x**_**Mn**_**x**_**Te NCs with spherical (a) and semispherical (b) confinements with radius R = 300 A and T = 4.8 K.** The other subband structure details are given in Figure 1.

Figure 1a,b shows the changes in the magnetic energy dispersions for the first few levels in *Pb*_1−*x*_*Mn*_*x*_*Se* dots with *R *= 300 A when the confinement is changed from spherical to semispherical. The broken symmetry induces stronger changes on the electron than on the hole energy dispersions by inducing anti-crossing regions. The exchange coupling affects mainly the conduction carrier dispersion. However, for *Pb*_1−*x*_*Mn*_*x*_*Te* dots with the same size *R*, shown in Figure 2a,b with both broken symmetry and exchange interaction, induce strong changes on both carrier magnetic dispersions but with the valence-band being more sensitive. The interplay between the usual Zeeman effect and the exchange interaction gives place to the crossing between spin-split levels at certain critical field, *B*_*c*_, as displayed in Figure 2 for both spherical and semispherical dot spatial confinements.

**Figure 3:**
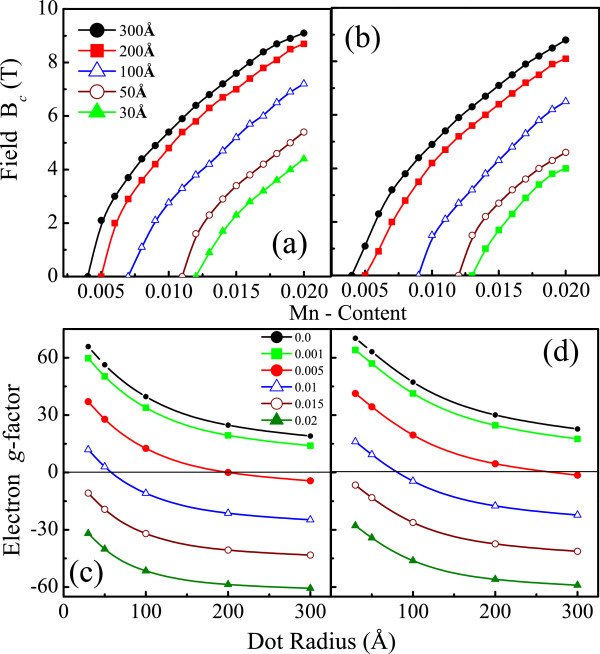
**Critical magnetic field as function of the Mn concentration for different Pb**_**1−x**_**Mn**_**x **_**Te NC radii Critical magnetic field as function of the Mn concentration for different Pb**_**1−x**_**Mn**_**x **_**Te NC radii (a,b); Landè g factor in the limit**B→0**as function of the NC radius for various Mn contents (c,d) and for the spherical (left panels) and semispherical confinements (right panels).**

Figure 3a,b shows that the critical field strength for *Pb*_1−*x*_*Mn*_*x*_*Te *dots, at a fixed temperature, increases with increasing Mn content for different dot sizes. Note that the smaller the dot size *R*, the larger the critical concentration *x*_*c *_where $B_{c}\Rightarrow 0$. For the limit B→0, we have calculated the Landè *g*-factor of the conduction band ground state of *Pb*_1−*x*_*Mn*_*x *_*Te* dots as *g*_*e*_*μ*_*B*_*B *=* E*(*e↑*,1/2,*N*)−*E*(*e↓*,−1/2,*N*), where $\mu_{B}=e\hbar /(2m_{0}c)$ is the Bohr magneton, *E*(*e↑*(*↓*),*F*_*z*_* N*) is the energy of the corresponding spin state, and *F*_*z *_=* L*_*z*_ + *J*_*z*_ is the *z*-component of total angular momentum **F **=** L** + **S**. The *g*_*e*_-values for *Pb*_1−*x*_*Mn*_*x*_*Te *dots as shown in Figure 3c,d displays similar behavior as reported in [9,10]*g*_*e*_(*BRx*)to approximately1/*R*.

As noted in Figure 3c,d, there are Mn concentration regions where the *g* factor becomes strictly positive or negative, independent of the confinement shape. For fixed dot radius, it is possible to predict the existence of a zero critical field value for a certain value *x*_*c *_for different dot and confinement geometries. For large dot sizes, a nonlinear increasing of *B*_*c*_ is observed for low values of *x* and a quasi-linear behavior otherwise.

In order to discuss the optical absorption spectrum, the probability for dipole-allowed optical transitions between single electron and hole states has to be evaluated in detail. Within the electrical dipole approximation, the oscillator strength is a linear combination of the matrix elements of the optical transitions, $M_{j,j^{\prime }}=\langle \psi_{j}|\hat{e}.\hat{P}|\psi_{j^{\prime }}\rangle =\langle f_{j}|f_{j^{\prime }}\rangle .\langle u_{j}|\hat{e}.\hat{P}|u_{j^{\prime }}\rangle +\langle u_{j}|u_{j^{\prime }}\rangle .\langle f_{j}|\hat{e}.\hat{P}|f_{j^{\prime }}\rangle$. Here, $\hat{e}$ is the light polarization vector, $\hat{P}$ is the momentum operator, _*fj*_and _*uj*_are the envelope and periodic Bloch functions at the *L* point for each involved carrier *j*, respectively. The second term on the right-hand side is responsible for intraband optical transitions, since 〈*u*_*j*_|*u*_*j′*_〉 =* δ*_*jj′*_. In this case the incident light couples, in the same band, state with different symmetries whenever the term $\langle f_{j}|\hat{e}.\hat{P}|f_{j^{\prime }}\rangle \neq 0$ for a given polarization. In our case the complete set of selection rules are obtained from the nonvanishing products of the matrix elements *I*_*e*,*h*_*δ*_*L*__*e*_,_*L*__*h*_*π*_*α*,*α′*_, where *π*_*α*,*α′*_ is the matrix of the parity operator, and *I*_*e*,*h*_ = 〈*f*_*e*,*α*_|*f*_*h*,*α*_〉 is the overlap integral of the electron-hole envelope functions allowed by the interband transition $\alpha \to \alpha^{\prime }$. The allowed transitions between states belonging to the Hilbert subspaces described by spinors (2) are determined from the angular dependence of the wave functions $f_{n,L}^{M}(r)$.

The corresponding selection rules for each optical transition in any polarization can be precisely obtained according to Kang et al. [2]. Due to the differences in the angular momenta *L* (symmetry and parity) of electron and hole spinor components, the allowed transitions occur only between initial (hole) and final (electron) states belonging to different Hilbert subspaces (I⇒II or II⇒I) for linear light polarization *Π*^*z*^ and for circular light polarization *σ*^±^. Moreover, the preservation of the total angular momentum *F*_*z*_, between initial and final states requires that *ΔM *= 0 for Voigt- *Π*^*z*^, and *ΔM *= ±1 for Faraday- *σ*^±^ geometry. For the circular polarization, the optical matrix element takes the form

(3) $$\langle \psi_{e,I}^{M_{e}}|{\hat{e}}^{\pm }\cdot \hat{P}|\psi_{h,\text{II}}^{M_{h}}\rangle =PF_{N_{e},M_{e}}^{N_{h},M_{h}}(I,\text{II})\delta_{M_{e},M_{h}\pm 1,}$$

where

(4) $$F_{N_{e},M_{e}}^{N_{h},M_{h}}(I,\text{II})=\underset{n,L\geq \left|M\right|}{\sum }\left[C_{n,\beta }^{e^{\pm }}C_{n,\beta }^{e^{\pm }}+C_{n,\beta }^{h^{\pm }}C_{n,\beta }^{h^{\pm }}\right],$$

with *β *= 2*L* + 1/2∓1/2. In the same way, the I→II transitions can be obtained by interchanging 2*L* + 1/2 ∓ 1/2 by 2*L* + 1/2 ± 1/2. The absorption coefficient can then be written as follows [7]:

(5) $$\begin{array}{lll} \;\alpha ({\hat{e}}^{\pm },\omega ) & =\alpha_{0}\underset{N_{e},N_{h},M}{\sum }\left(\frac{\Gamma }{\Pi }\right)\; & \;\\ & \;\times \left(\frac{{\left|F_{N_{e},M}^{N_{h},M\pm 1}(I,\text{II})\right|}^{2}}{{\left[E_{N_{e},M}(I)-E_{N_{h},M\pm 1}(\text{II})-\hbar \omega \right]}^{2}+\Gamma^{2}}\right)\; & \;\\ & \;+\left(\frac{{\left|F_{N_{e},M}^{N_{h},M\mp 1}(I,\text{II})\right|}^{2}}{{\left[E_{N_{e},M}(\text{II})-E_{N_{h},M\mp 1}(I)-\hbar \omega \right]}^{2}+\Gamma^{2}}\right),\; \end{array}$$

where *α*_0_ is a magnitude which includes the bulk *P* parameter and the dielectric constant. The material parameters can be found in [4,5]. For the linear light polarization *Π*^*z*^, the optical matrix element becomes

(6) $$\langle \psi_{e,I}^{M_{e}}|{\hat{e}}^{\pm }\cdot \hat{P}|\psi_{h,\text{II}}^{M_{h}}\rangle =PV_{N_{e},M_{e}}^{N_{h},M_{h}}(I,\text{II})\delta_{M_{e},M_{h}\pm 1,}$$

where

(7) $$V_{N_{e},M_{e}}^{N_{h},M_{h}}(I,\text{II})=\underset{n,L\geq \left|M\right|}{\sum }\left[C_{n,\beta }^{e^{+}}C_{n,\beta }^{h^{+}}+C_{n,\beta }^{e^{-}}C_{n,\beta }^{h^{-}}\right],$$

and the related absorption coefficient turns

(8) $$\begin{array}{lll} \;\alpha ({\hat{e}}^{z},\omega ) & =\alpha_{0}\underset{N_{e},N_{h},M}{\sum }\left(\frac{\Gamma }{\Pi }\right)\; & \;\\ & \;\times \left(\frac{{\left|V_{N_{e},M}^{N_{h},M}(I,\text{II})\right|}^{2}}{{\left[E_{N_{e},M}(I)-E_{N_{h},M}(\text{II})-\hbar \omega \right]}^{2}+\Gamma^{2}}\right)\; & \;\\ & \;+\left(\frac{{\left|V_{N_{e},M}^{N_{h},M}(I,\text{II})\right|}^{2}}{{\left[E_{N_{e},M}(\text{II})-E_{N_{h},M}(I)-\hbar \omega \right]}^{2}+\Gamma^{2}}\right).\; \end{array}$$

In the case of semispherical geometry, the selection rules for the circular light polarization are the same as for the spherical case; meanwhile, for the linear light polarization, these allow transitions within the same subspace due to the parities of the components of the wave functions in the subspaces.

**Figure 4:**
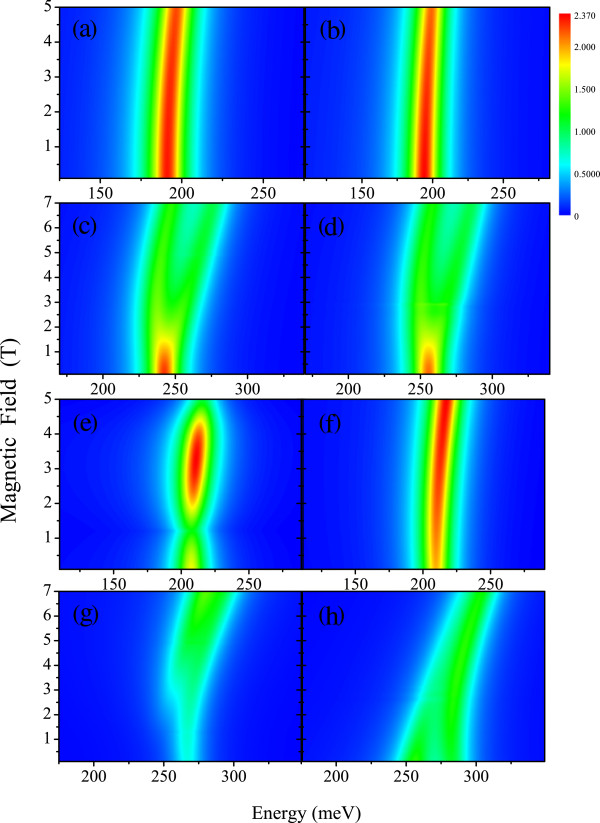
**Interband absorption spectra as function of magnetic field for polarization *Π***^**z **^**(a-d) and *σ***^**+ **^**(e-h) Pb**_**0.99**_**Mn**_**0.01**_**Se NCs with spherical (a,e) and semispherical (b,f) confinements and Pb**_**0.99**_**Mn**_**0.01**_**Te NC with spherical (c,g) and semispherical (d,h) confinements.** The same parameters were as referred in Figures 1 and 2.

**Figure 5:**
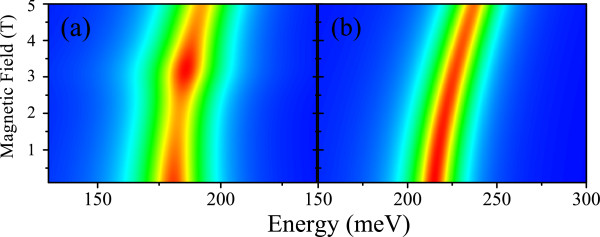
**Interband absorption spectra as function of magnetic field for polarization *σ***^**+ **^**of PbSe (a) and PbTe NCs (b) with spherical confinement of *R *= 300 A and temperatures 1.8 and 4.8 K, respectively.**

The excitonic resonances for *Π*^*z *^and *σ*^+^ , calculated as a function of the magnetic field for each Mn-doped lead-salt dot and confinements, are shown in Figure 4a,b,c,d,e,f,g,h. In Figure 5, we displayed the corresponding excitonic resonances for *σ*^+^ of the reference samples (without Mn doping) for spherical confinement. Comparing Figures 4e and 5a and Figures 4g and 5b, we confirm that the effect of Mn doping on the absorption spectra is stronger on the bandgap renormalization than on the subband levels in the doped salt-selenide unlike the salt-telluride, where the Mn presence strongly modifies all the band structure [11,12]. The resonant transitions shown in Figure 5a,b involve just the conduction band ground state of spherical and semispherical PbMnSe dots. The corresponding spectra for PbMnTe, shown in Figure 4c,d, correspond to the transitions to the first crossing conduction band levels. Figure 4d displays an absorption bottleneck due to the level crossing (see Figure 2a,b) for PbMnSe spherical dots. Another absorption quenching appears at *B *= 1.2T in Figure 4e caused by the character admixture close to a level crossing. In turn, Figure 4f displays a single transition to the conduction band ground state. In Figures 4g,h two transitions appear that fade-off for lower and higher fields, respectively. This effect is produced by the modulation of the oscillator strength. For small nanocrystal size, the spectra will show quantitative variation due to the effective gap modulation and the subsequent weakening of the intersubband coupling.

## Conclusions

Summarizing, we have investigated the electronic and magneto-optical properties of *Pb*_1−*x*_*Mn*_*x *_*Se* and *Pb*_1−*x*_*Mn*_*x*_* Te* semimagnetic dots by taking advantage of their strong sensitivity to spatial confinement asymmetry and properties induced by the Mn doping. We have shown the appearance of the critical phenomena as the spin level crossing for certain concentration of Mn on the *Pb*_1−*x*_*Mn*_*x *_*Te *and the modulation of the optical absorption controlled by field B and confinement anisotropy. Subtle effects of Mn content variation were predicted for the energy spectra of the *Pb*_1−*x*_*Mn*_*x *_*Se* dots, whereas important consequences are expected for *Pb*_1−*x*_*Mn*_*x *_*Te *dots. We believe that these results may stimulate research groups working on these important materials to explore device applications working on the wide spectral range.

## Competing interests

The authors declare that they have no competing interests.

## Authors’ contributions

SJP carried out the calculation of the band structure and absorption spectra and participated in the study of the electronic and magneto-optical properties. LVL, VLR and GEM participated in the design of the problem, and its study and coordination. AMA conceived of the study and participated in the design of the problem and first stages of calculation. All authors read and approved the final manuscript.
